# MicroRNAs as a Novel Tool in the Diagnosis of Liver Lipid Dysregulation and Fatty Liver Disease

**DOI:** 10.3390/molecules24020230

**Published:** 2019-01-09

**Authors:** Jingwei Yu, Jun Peng, Zhilin Luan, Feng Zheng, Wen Su

**Affiliations:** 1 Shenzhen University Medical Center, Shenzhen University Health Science Center, Shenzhen 518060, China; brent1008@gmail.com (J.Y.); fkuejun3092@163.com (J.P.); 2 Department of Biology, Guangdong Pharmaceutical University, Guangzhou 510006, China; 3 Advanced Institute for Medical Sciences, Dalian Medical University, Dalian, Liaoning 116044, China; luanzl@dmu.edu.cn (Z.L.); fzheng63@163.com (F.Z.)

**Keywords:** microRNA, fatty liver disease, lipogenesis

## Abstract

In recent years, metabolic disorder, especially fatty liver disease, has been considered a major challenge to global health. The attention of researchers focused on expanding knowledge of the regulation mechanism behind these diseases and towards the new diagnostics tools and treatments. The pathophysiology of the fatty liver disease is undoubtedly complex. Abnormal hepatic lipid accumulation is a major symptom of most metabolic diseases. Therefore, the identification of novel regulation factors of lipid metabolism is important and meaningful. As a new diagnostic tool, the function of microRNAs during fatty liver disease has recently come into notice in biological research. Accumulating evidence supports the influence of miRNAs in lipid metabolism. In this review, we discuss the potential role of miRNAs in liver lipid metabolism and the pathogenesis of fatty liver disease.

## 1. Introduction

In vertebrates, the liver is a vital gland tissue that plays several major roles in regulating the metabolism of carbohydrates, proteins, amino acids, and lipids. The majority of lipoproteins, such as very low-density lipoprotein (VLDL) and nascent high-density lipoprotein (HDL), are synthesized in the liver. Meanwhile, the liver is also an important accessory digestive organ, producing bile acids that aid in lipids digestion [1,2,3,4]. Various molecules have been proven to playing important regulatory roles in the lipid metabolism, including several nuclear transcription factors: liver X receptors (LXRs), sterol regulatory element-binding proteins (SREBPs) and farnesoid X receptors (FXRs). These nuclear receptors are involved in the regulation network of the lipid metabolism along with other molecules, including various miRNAs.

**Figure 1:**
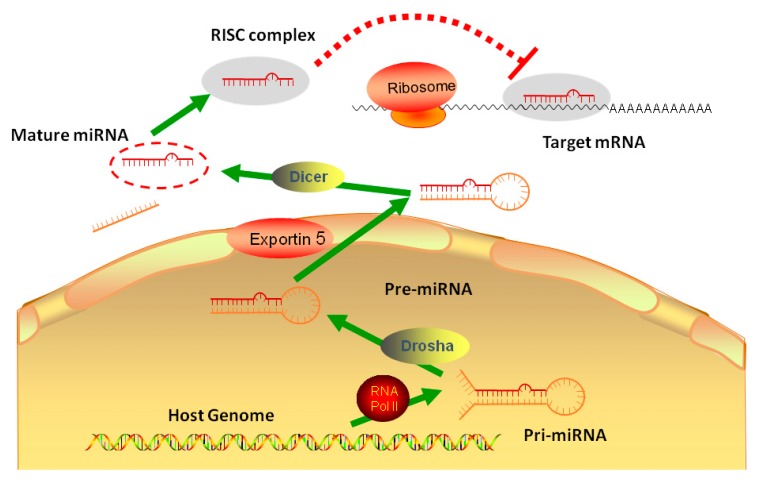
The biogenesis and regulation of microRNA: One miRNA is initially transcribed by RNA polymerase II (RNA Pol II) as part of one arm of a several hundred nucleotide-long primary miRNA (pri-miRNA). The pri-miRNA is cleaved by Drosha, a Class 2 ribonuclease III enzyme, to produce a characteristic stem-loop structure of about 70 base pairs long, known as a pre-miRNA. Endoribonuclease Dicer cleaves pre-microRNA (pre-miRNA) into short single-stranded RNA fragments called mature miRNA in cytoplasm. Mature miRNA form RISC complex to combine the target mRNA.

MicroRNAs (miRNAs) are small endogenous non-coding RNA molecules, generally about 18-22nt in length, which act as regulators of protein expression. After miRNAs were first discovered as a regulator in the development process of *Caenorhabditis elegans*, numerous miRNAs have subsequently been demonstrated to carry broad influences over a wide range of biological processes. In the mammalian canonical miRNA pathway, miRNA genes are usually transcribed by RNA polymerase II in the nucleus to produce long-chain primary miRNA (pri-miRNA) transcripts, which are subsequently processed to form the stem-loop hairpin structures of precursor miRNA (pre-miRNA) by RNase III enzyme Drosha. Then, pre-miRNAs are transported from the nucleus to the cytosol through exportin-5 and are subsequently spliced to mature double-stranded miRNA by Dicer, an enzyme belonging to the RNase III family. The mature miRNAs can complex with argonaut proteins 2 (Ago2) to form RNA-induced Silencing complex (RISC). The RISC-attached mature miRNAs then hybridize to a complementary sequence in the three prime untranslated regions (3′ UTR) of specific mRNA targets, and facilitates the post-transcriptional regulation (Figure 1). The regulatory mechanisms induced by miRNA binding have not been fully illustrated but seem to involve translation repression, deadenylation, and degradation of the target mRNAs. Each miRNA is predicted to target several genes, and 3′ UTR of each specific mRNA can hold putative target sites for many miRNAs [4,5,6]. miRNAs have recently been reported to present another regulatory layer overlaying and intersecting with a transcriptional control mechanism in keeping metabolic homeostasis [7,8,9]. Furthermore, our understanding for miRNA was expanded from bench to bedsides, miRNAs shows the potential of diagnostic and treatment for many diseases, including liver disease.

In this review, we discuss recent advances in our understanding of the emerging roles of miRNAs in guiding lipid homeostasis in the liver with emphasis on the progression of fatty liver disease.

## 2. Overview of Liver Lipid Metabolism

Lipids have diverse biological functions serving as crucial structural components of cell membranes, sources of important energy storage, and as signaling molecules (such as steroid hormones). Lipid metabolism involves multiple pathway, the core elements of which the present discussion will focus on include those involving triglycerides and fatty acids [10,11].

**Figure 2:**
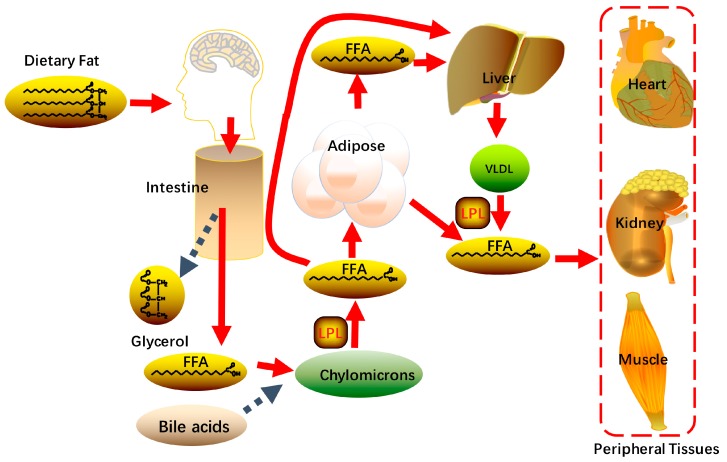
Digestion and metabolism of dietary lipids: Dietary fat is hydrolyzed to glycerol and free fatty acids (FFA) in intestine. Most of the FFA are delivered to adipose tissue for storage; some are transported to the liver for lipid synthesis. Excessive free fatty acids in the peripheral circulation lead to lipid ectopic deposition in tissues.

Food is a major source of daily fat, with dietary fat (mainly triglyceride) being hydrolyzed to free fatty acids and glycerol in the intestine via pancreatic lipases and varied enzymes carried by the gut microbiota. Apart from short chain fatty acids that enter the circulation directly, the absorbance of most dietary fatty acids from the small intestine depends on the presence of sufficient bile acids. Bile acid-emulsified fatty acids can be used for triglycerides synthesis and then enter the circulation as lipoprotein particles called chylomicrons via the lymphatic system. The chylomicron-attached triglycerides can be attacked by lipoprotein lipase and release free fatty acids (FFAs) at the endothelial surface of capillaries [12]. The resulting fatty acids (~70%) are delivered to adipose tissue and stored as lipid droplets, a portion of which can be stored as energy fuel, while the remainder transferred the liver (Figure 2).

Free fatty acids are largely bound to albumin in plasma for its hydrophobicity, fatty acids uptake into the liver thereby requires the dissociation of FFAs from albumin [9,11,13,14,15]. While the members of fatty acid transport protein (FATP) family, like FATP2, FATP5 and cluster of differentiation 36 (CD36) then mediate transportation of FFAs across the plasma membrane [16,17]. In mammalian cells, FATP2 is enriched in liver and kidney whereas FATP5 is a hepatic-specific isoform. CD36 is widely expressed in various tissues and cells while lowly expressed in hepatocytes. Deletion of CD36 does not influence the development of hepatic steatosis in mice, illustrating that uptake of fatty acids in hepatocytes is mainly dependent on FATPs [18,19].

Once uptaken into hepatocyte cytosol, FFAs are used to synthesize fatty acyl-CoAs through the fatty acyl-CoA synthetases (ACSs). However, previous studies have demonstrated the FATPs themselves exhibit both long chain and very long chain fatty acyl-CoA synthetase activity [20,21]. Fatty acyl-CoA is a temporary compound formed by adding coenzyme (CoA) to the end of fatty acids, which will quickly undergo β-oxidation to break down fatty acids and generate ATP or be incorporated into triglycerides [22,23]. Fatty acid oxidation occurs mainly in mitochondria, and partly in peroxisomes or microsomes. Since no ACSs exists in the mitochondrial complex, fatty acids traverse mitochondrial membranes in the form of fatty acyl-CoA. Short-chain fatty acyl-CoAs can simply diffuse across the inner mitochondrial membrane, while long-chain fatty acyl-CoAs must be coupled with free carnitine and then converted into acyl-carnitine by the carnitine palmitoyltransferase 1 (CPT 1) in the outer mitochondrial membrane [11,19,24,25]. Fatty acyl-CoAs are subsequently cleaved into two carbon segments to synthesize acetyl-CoA in each β-oxidation cycle, along with abundant production of ATP. Acetyl-CoA as the final product of β-oxidation take part in a series of biochemical reaction, like the tricarboxylic acid (TCA) cycle and also used back to lipid de novo synthesis [26,27,28].

**Figure 3:**
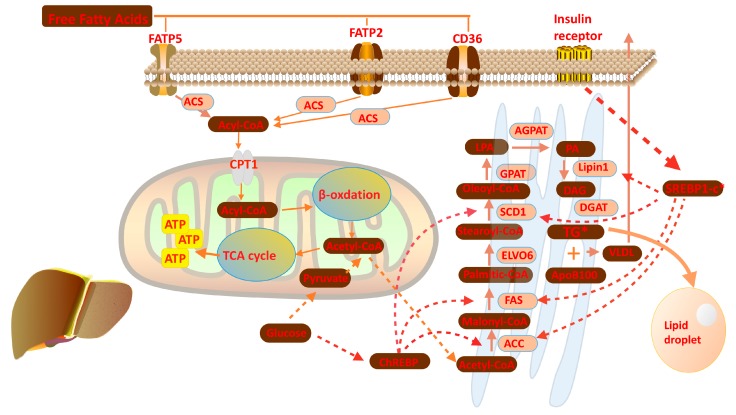
Overview of the lipid metabolism in the liver: Free fatty acids are used to synthesize acyl-CoAs after uptake into hepatocyte cell under the help of FATP family; Acyl-CoA transfer to mitochondria to participate β-oxidation, which finally produce acetyl-CoA; Acetyl-CoA are used to synthesis lipid in smooth endoplasmic reticulum.

Except being the fuel to provide energy, exogenous fatty acids are rapidly assimilated into neutral and polar lipids such as glycerolipids, sterols, and glycerophospholipids. Otherwise, in the setting of excess carbohydrates intake, hepatic de novo lipogenesis (DNL) is triggered in the liver by extensive glucose or other factors. After uptake into the liver, glucose is converted to acetyl-CoA via glycolysis and oxidation of pyruvate. Acetyl-CoA carboxylase (ACC) catalyzes the formation of malonyl-CoA from acetyl-CoA; then this newly synthesized product is used to assemble palmitic acid by joining with acetyl-CoA by fatty acid synthase (FAS) [29,30,31]. Palmitic acids are elongated and desaturated to generate oleoyl-CoA through stearoyl-CoA desaturase (SCD1) and long chain fatty acid elongase 6 (ELOVL6). Oleoyl-CoAs are substrates for glycerol-3-phosphate acyltransferase (GPAT) to catalyze the formation of lysophosphatidic acids (LPA) [32]. 1-acylglycerol-3-phosphate sequentially converts LPAs to phosphatidic acids (PA), which then are processed to diacylglycerols (DAG) by lipin1. DAGs will be used to form triglycerides through acyl-CoA: diacylglycerol acyltransferase (DGAT) (Figure 3). Hepatic de novo lipogenesis is regulated primarily at the transcriptional level [33,34]. Under normal dietary conditions, the accumulation of circulating glucose and insulin facilitate lipogenesis in order to maintain the homeostasis of glucose, which is related to the regulation of two transcription factors: carbohydrate response element binding protein (ChREBP) and sSREBP1c. Insulin physiologically stimulates the SREBP1c expression and finally results in upregulation of several lipogenic genes, such as FAS, ACC, SCD1 and lipin 1 [35,36,37]. The mechanism by which insulin facilitate SREBP1c expression is phosphoinositide 3-kinase (PI3K) dependent and relies on the participation of LXRs. As a nuclear receptor, LXR mediated transactivation requires the formation of a heterodimer complex with retinoid X receptor (RXR) and binding its ligands [38,39,40]. The complex binds to the LXR response element, usually located upstream of target genes in their promoter regions. ChREBP also has important functions on lipogenesis triggered by glucose within the liver. ChREBP is a glucose-sensitive transcription factor that acts on lipogenesis independently or in conjunction with LXR-RXR complex [41,42].

The lipids can incorporate into very low-density lipoprotein (VLDL) particles and transport from the liver into peripheral tissue. VLDL is a triglyceride-rich lipoprotein which is synthesized in the liver, and the mechanism of VLDL synthesis and secretion is well known [43,44,45]. The VLDL particle consists of a hydrophobic core composed triglycerides and cholesterol. The long polypeptide apolipoprotein B100 (ApoB 100) is a critical factor when the VLDL particle is assembling, which is translocated onto the surface to stabilized VLDL structure [46]. Once mature VLDL particles are release into circulation from hepatocytes, they will contact with lipoprotein lipase (LPL) in capillary beds of peripheral tissues, such as cardiac, skeletal muscle and adipose tissue. The triglycerides contained in VLDL are hydrolyzed to FFAs that provide energy fuel or storage in different tissues outside the liver [47]. Serving as an important traffic hub for triglycerides and fatty acids in the body, the liver is a critical organ for maintaining the whole body’s lipid and glucose homeostasis. Under physiological conditions, these metabolic pathways described above is to keep homeostasis of intracellular FFA and acyl-CoA.

## 3. miRNAs in Lipid Metabolism

miRNAs play a significant role in regulating many facets of liver lipid metabolism by targeting varied transcripts across different cell types. For instance, many miRNAs take part in adipogenesis. miR-143 is a well-documented miRNA shown to participate in human adipocyte differentiation, with overexpression of miR-143 accelerating the differentiation process of murine preadipocytes, and specific inhibition of miR-143 blunting adipogenesis [48,49,50]. In recent studies, miR-204, miR-200c, miR-141, and miR-439, are also been reported to participate in early adipocyte cell fate determination, while others, including miR-27a, miR-378, miR-130 and etc., are involved in the terminal stage of adipocyte differentiation [51,52].

The excessive lipid accumulation in adipose tissue leads to obesity and finally contributes to metabolic syndrome. In previous studies, increased expression of miR-335 was found both in liver and white adipose tissues of obese mice, such as leptin-deficient (*ob/ob*) mice, leptin receptor-deficient (*db/db*) mice, compared to normal mice [51,52,53]. miR-335 also influences lipid metabolism and may participate in the differentiation of human mesenchymal stem cells, which exhibit adipogenic potential [54,55]. Similarly, expression of miR-335 is upregulated during mouse preadipocyte differentiation [56]. However, the molecular basis for miR-335 in the regulation of adipogenesis and lipid metabolism remains elusive.

In addition, miRNAs may also regulate the lipid metabolism in the liver. There are 150 miRNAs which are upregulated in mice fed with high-fat diet. Many of these miRNAs have been identified to regulate metabolic processes in the liver, although their roles on fatty liver pathogenesis remain to be determined [57,58].

As the first identified miRNA participating in lipid metabolism, miR-122 is a liver-specific and liver-enriched miRNA, accounting for nearly 70% of total hepatic miRNA expression [59,60]. General knockout or conditionally hepatic knockdown of miR-122 significantly decreases serum triglyceride and total cholesterol levels. Similarly, after blocking biological function of endogenous miR-122 via complementary antisense-locked nucleic acid, there was a significant reduction (~30%) of circulating cholesterol levels in mice [61]. Consistently, other studies revealed that a set of cholesterol biosynthesis genes were down-regulated by miR-122 by an indirectly regulation manner, including 3-hydroxy-3-methylglutaryl-coenzyme A reductase (HMGCR) and microsomal TG transfer protein (MTTP), 3-hydroxy-3-methylglutaryl-coenzyme A synthase 1 (HMGCS1) [17,62,63,64].

However, miR-122 has been shown to induce the expression of genes involved in de novo lipogenesis including SREBP1-c, DGAT2, FAS, and ACC1. Interestingly, miR-122 may also interact with other miRNAs during the development of fatty liver disease. For instance, overexpression of miR-370 in HepG2 cells stimulates the expression of lipogenic genes FAS and ACC1 through modulation of SREBP-1c expression [19]. However, silencing of miR-122 in HepG2 cells abolishes miR-370 mediated activation of SREBP-1c. In addition, transfection of HepG2 cells with miR-370 induces up-regulation of miR-122, and knockdown miR-370 in vivo leads to miR-122 down-regulation. Therefore, these findings suggest that miR-370 regulates expression of genes involved in lipid metabolism via miR-122 [65,66].

Another important miRNA, miR-33, which is extensively involved in the regulation of liver lipid metabolism and shows great therapeutic potential to fatty liver disease [7,67]. The miR-33 family comprises two members, miR-33a and miR-33b. In humans, miR-33a and miR-33b are located in the intronic regions of SREBP2 and SREBP1, respectively [68,69,70]. As mentioned above, SREBPs are key regulators of cholesterol and lipids synthesis, accompany with their transcripts, miR-33 can translate and participate in the regulation of similar physiological processes. Blocking the function of miR-33 in vivo increases the circulation HDL concentrations through targeting adenosine triphosphate-binding cassette transporter A1 (ABCA1) and adenosine triphosphate-binding cassette transporter G1 (ABCG1), and thus further suppresses cholesterol efflux to apolipoprotein A1 (ApoA1) or nascent HDL [67,71]. Besides the role in cholesterol metabolism, miR-33 also blunts fatty acid oxidation and regulates insulin signaling [72]. Inhibition of miR-33 reduced the circulation levels of VLDL, by increasing the expression of key enzymes involved in fatty acid oxidation, including Carnitine Palmitoyltransferase 1A (CPT1A), Hydroxylacyl-CoA Dehydrogenase/3 Ketoacyl-CoA Thiolase (HADHB), Carnitine *O*-Octanoyltransferase (CROT) and so on. Consistently, overexpression of miR-33 in hepatocytes can lead to the significant accumulation of triglycerides in the cytoplasm, accompanied with inhibition of β-oxidation.

Additionally, it has been demonstrated that the miRNAs, miR-27a and miR-27b can regulate the adipogenesis through targeting of Retinoid X receptor alpha (RXRα) and PPARγ. Overexpression of miR-27b stimulates lipolysis and lipids secretion from cells in the form of glycerol or free fatty acids. On the other hand, increased miR-27a represses several lipid metabolic genes, such as FAS, SREBP-1, peroxisome proliferator-activated receptor-α (PPARα) [57]. By high-throughput small RNA sequencing and consequent in silico analysis, miR-27b is considered as a regulatory hub in lipid metabolism of human hepatocytes (HuH7 cells). Hepatic miR-27b is responsive to lipid levels and is predicted to affect 27 lipid metabolism-related target genes. Some of these targets, such as angiopoietin-like 3 (ANGPTL3), glycerol-3-phosphate acyltransferase (GPAM) and N-deacetylase-N-sulfotransferase (NDST1) were already validated by experiments for both functional importance as well as direct interaction [73]. For instance, GPAM is highly expressed in liver, and plays role in catalyzing the first committed step in DNL as mentioned; overexpression of GPAM causes steatosis and hepatosis [74]. The plasma level of ANGPTL3, secreted from the liver, is closely correlated with the progression of dyslipidemia and atherosclerosis. Accompanied by this, hepatic miR-27b is upregulated in ApoE knockout induced dyslipidemia animal mode [75,76].

## 4. miRNAs in Nonalcoholic Fatty Liver Disease

Nonalcoholic fatty liver disease (NAFLD) is the most common chronic liver disease in modern societies, and it is also considered a manifestation of metabolic syndrome. established risk factors associated with NAFLD and more progressive diseases include obesity (central), hypertension, dyslipidemia, type 2 diabetes and metabolic syndrome [77]. The hallmark of NAFLD is hepatic lipid ectopic accumulation (conventionally set as more than 5% by weight) in the absence of excessive alcohol consumption and other forms of chronic liver diseases [78]. The term NAFLD comprises a serial of progressions, from simple steatosis over non-alcoholic steatohepatitis (NASH) to cirrhosis and hepatocellular carcinoma (HCC). Despite the high prevalence of NAFLD in the general population, the majority of patients merely have simple steatosis, and experience similar life expectancy and transaminase levels as the general population; only 5–10% of patients who are diagnosed with NAFLD will progress to NASH and 30% of the NASH patients will eventually develop liver fibrosis [79,80].

Although the pathogenesis of NAFLD is incompletely understood, there is a “two-hit hypothesis” proposed to explain the sequential evolution from steatosis to steatohepatitis or advanced NASH. The first “hit” is insulin resistance, which is induced by dietary habits, together with genetic factors. Insulin resistance of the white adipose tissue results in increased fatty acid flux to the liver with subsequent ectopic hepatic fat deposition and causes the liver to become more susceptible to injury [81]. The second “hit” is from oxidative stress, increased cytokine and activated inflammation cascades, finally resulting in NAFLD [82]. However, this theory has been extended to a new concept termed “multiple hit” hypothesis later.

The new theory deems multiple insults effect together on genetically predisposed subjects to induce NAFLD and introduce another consideration of NAFLD pathogenesis [83]. In addition to insulin resistance, the multiple hits include more factors such as circulation adipokines, nutritional factors, intestinal microbiota, genetic and epigenetic factors. Clinical investigations showed that epigenetic modification occurred during NASH development. Epigenetic processes, including DNA methylation, histones modifications and the activity of miRNAs, could regulate gene expression at the transcriptional level without DNA sequence alteration. DNA methylation is already identified as one of the crucial determinants during progression from steatosis to NASH and is affected by the concentration of fundamental methyl donors in dietary, such as betaine, choline, and folate [84].

To date, a number of recent studies conducted both in vitro and in vivo have illustrated that miRNAs could regulate epigenetic mechanisms of gene expression, which is not only involved in the regulation of cellular growth and differentiation even in the control of energy balance and hepatic lipid metabolism. The emerging roles of miRNAs on adipocytes differentiation, insulin resistance, hepatic lipid metabolism, and inflammation implicate the potential relationship between miRNAs and NAFLD pathogenesis. Many studies have been designed to dig into the regulation of miRNAs on NAFLD.

In a previous study, the circulation levels of miRNAs are altered in different stages of NAFLD, and some specific miRNAs are correlated with the pathogenesis of NAFLD. By using Sprague-Dawley (SD) rats to generate NAFLD animal model, researchers characterized 58 up-regulated miRNAs and 51 down-regulated miRNAs in different stages of NAFLD [85]. Several members of these detected miRNAs, like miR-16, miR-29c, and miR-122, are reported to have the impact on many biological activities [86]. For instance, miR-16 is gradually increased along with NAFLD pathogenesis, which is a known apoptosis regulation factor. Therefore, miR-16 may have the potential to regulate hepatocyte apoptosis during NAFLD pathogenesis [87]. While miR-29c and miR-122 have been proved that they could regulate insulin resistance and lipid metabolism, which implicated their possible roles in the development of NAFLD [88]. In fact, serum-miR-29c and miR-122 are continually increased throughout the progression of NAFLD in rats [89].

Likewise, in another study with western type diet-induced NAFLD in LDLR knockout mice, hepatic miRNA profile was significantly altered during NAFLD progression. The transcriptome data revealed that miR-216 and miR-302a could play an important role in fatty liver development. Especially, miR-302a is predicted to target ELOVL6 which is involved in the elongation of palmitate to stearate. Meanwhile, the decrease in miR-302a expression is associated with a parallel increase in the expression of ELOVL6 [90]. This evidence indicates that miR-302a could regulate the lipid synthesis during NAFLD development.

miR-34a, another miRNA related to NAFLD which is increased in the serum of patients with NAFLD [91,92]. It has been reported that miR-34a targets sirtuin-1 (SIRT1) and blunts its biological function. SIRT1 is an activator of Adenosine 5′-monophosphate (AMP)-activated protein kinase (AMPK) pathway which inhibits hepatocyte lipid accumulation [93]. Recently, a new target of miR-34a, PPARα, have been identified [94]. PPARα is crucial to regulating lipid transport and metabolism, especially playing an important role on the mitochondrial β-oxidation pathway. The target genes of PPARα include fatty acid (FA)-metabolizing enzymes, such as mitochondrial FA oxidation related enzymes, the majority of which exist in the liver [95,96]. Both mRNA and protein levels of PPARα were directly downregulated by miR-34a in (mouse) liver, which could be rescued by inhibiting miR-34a expression. Consistent with this, knockdown of miR-34a markedly attenuated the FFA induced lipid accumulation in hepatocytes in vitro. Meanwhile, administrating miR-34a inhibitor through vein injection could alleviate high-fat-diet induced hepatic steatosis. In this process, increased PPARα expression not only stimulated the AMPK signaling but also activated fatty acids β-oxidation-related genes, which could further decrease the lipid accumulation in liver [94].

miR-24 is also found that it’s upregulated in the liver of mice fed with high-fat diet [97]. Moreover, miR-24 was upregulated in FA treated HepG2 cells and primary human hepatocytes [98]. Bioinformatic and experimental results prove that miR-24 could directly bind the 3′ UTR region of insulin-induced gene 1 (*Insig1*), an inhibitor of lipogenesis. Silencing of endogenous miR-24 leads to up-regulation of *Insig1* in vitro and subsequently blocks the hepatic lipid accumulation. An (in vivo) study also showed that miR-24 overexpression facilitates SREBP-1c processing and further upregulates the expression of lipogenic genes, through targeting *Insig1* [98]. These researches suggest the potential role of miR-24 in hepatic lipid accumulation. Thus, miR-24 may become a potential therapeutic target for the obesity-related NAFLD.

Recently, some miRNAs were identified that their expression level in peripheral blood and liver were both correlated with the development of NAFLD, including miR-122 and miR-21 [99,100]. As we discussed above, nearly 70% of liver miRNAs is miR-122, and the function of miR-122 in lipid metabolism has been well demonstrated. For miR-21, its expression is lower in the serum but higher in the liver in NAFLD patients compared with healthy people, although the cause of its expression difference between serum and liver remains unknown [101]. However, Li and his colleagues observed that expression level miR-21 was gradually declined in the livers of diet-induced NASH mice compared with the control group, with the development of disease [53]. Transfecting HepG2 cells with miR-21 mimics suppressed the levels of triglyceride (TG), total cholesterol (TC) and free cholesterol (FC) in cells. Meanwhile, HMGCR, the rate-limiting gene of cholesterol biosynthesis was down-regulated by miR-21 mimics. Through in silico analysis and experimental validation, HMGCR is identified to be a direct target of miR-21. Inhibition of miR-21 led to increased expression of HMGCR. Taken together, these results demonstrate that miR-21 regulate liver TG and cholesterol metabolism through directly targeting HMGCR [102].

Another NAFLD associated miRNA is miR-149. Its expression level is elevated in FA treated HepG2 cells, and also upregulated in NAFLD animal model [103]. In addition, the absence of miR-149 could block the lipogenesis in FA-treated HepG2 cells, and transfection of miR-149 mimics induces lipid accumulation in normal culture condition without the addition of excessive exogenous fatty acids. Through bioinformatics analysis, fibroblast growth factor 21 (FGF-21) was identified as the target gene of miR-149 [103]. FGF-21 is a member of the fibroblast growth factors, which is highly expressed in muscle, white adipose tissue and liver. FGF-21 plays critical regulatory roles on lipid metabolism and benefit improvement of NAFLD [104,105]. Previous studies have illustrated that miR-149 negatively regulates protein translation of FGF-21, and subsequently promotes lipogenesis in HepG2 cells. In consideration of over-expression of FGF-21 in vivo has been shown to ameliorate fatty liver [105], pharmacological inhibition of endogenous miR-149 might be a new therapeutic strategy for NAFLD.

In addition to excessive lipid accumulation, hepatocyte inflammation and apoptosis are important pathological elements for the progression of NAFLD [83]. miRNAs could also participate in the progression of steatohepatitis, such as miR-10b, miR-144, miR-155, and miR-146b [106,107,108]. It’s reported that miR-10b could increase the lipid contents in steatosis L02 cells by targeting PPAR-α. In particular, miR-155 is considered one of the important regulators of inflammation, which influences both innate and adaptive immunity [109]. Feeding with Methionine-choline-deficient (MCD) diet induces steatohepatitis in mice and increases miR-155 in the entire liver in the meantime [110,111]. Early research indicates that miR-155 is triggered by Toll-like receptor activation, which subsequently increases the translation of tumor necrosis factor alpha (TNFα), a key inflammatory cytokine involved in the progression of steatohepatitis [112]. More interestingly, it has been confirmed that LXRα is a direct target of miR-155 [113]. Higher activity of LXRα induces the expression of SREBP-1c and subsequently facilitates lipogenesis and lipids accumulation in the liver [113]. Consistently, liver steatosis is improved and the expression of genes involved in lipogenesis is decreased in miR-155 knockout mice fed with MCD, compared with the wild type mice [111]. Clinical studies also found that miR-155 is downregulated in the liver and peripheral circulation of NAFLD patients [110], This evidence suggested that miR-155 could participate in the regulation of lipid metabolism and inflammation in the progression of steatohepatitis.

## 5. miRNAs in Alcohol and Virus-Induced Fatty Liver

Long-term alcohol overconsumption can induce alcoholic liver disease (ALD). The manifestations include fatty liver, alcoholic hepatitis, and liver fibrosis or cirrhosis. Moreover, patients with ALD generally simultaneously suffer from nonalcoholic fatty liver disease, or chronic viral hepatitis.

The liver is the primary organ for alcohol metabolism, while hepatocytes produce most of the alcohol metabolizing enzymes, like majority of alcohol dehydrogenases (ADHs) and cytochrome P450 2E1 (CYP2E1) which are expressed in the liver [114,115]. Meanwhile, steatosis is the most common response of the liver to chronic alcohol consumption [116]. Steatosis can occur in any individual who administrates excess alcohol over a long period of time, and this process is transient and reversible [117]. As mentioned before, miR-155 is harmful to non-alcoholic liver steatosis and fibrosis. Coincidentally, the miR-155 expression is also induced in the liver of ALD mouse model, especially in hepatocytes and Kupffer cells [118,119]. Meanwhile, a serial of lipid metabolism-related genes are downregulated in miR-155 knockout MCD fed mice, including ADRP, DGAT2, CPT1a, and PPARα [111], which suggests that miR-155 may be involved in hepatic lipid metabolism in alcoholic fatty disease.

The presence of alcohol in the liver can accelerate hepatic lipid synthesis and weaken fatty acid oxidation. Previous studies have found that alcohol activates hepatic SREPB-1 processing, thereby stimulates hepatocytes transferring from lipid consumption to lipid storage [120]. It’s well known that SIRT1 plays a crucial role in lipid metabolism by deacetylation of modified lysine residues on its target gene like SREBP-1. Activation of SITR1 inhibits the gene expression of SREBP-1 [121]. In recent studies, alcohol has been proved to suppress the activity of SIRT1 in the liver, which subsequently induces SREBP-1 signaling and lipogenesis [122]. The evidence is now emerging to suggest that hepatic steatosis induced by chronic alcohol exposure is mainly mediated by SIRT1 [123].

miR-217 should be specially mentioned due to its roles on alcohol-induced hepatic steatosis. The expression of miR-217 in hepatocytes is dramatically upregulated by alcohol treatment either in vivo or in vitro studies [124]. It has been demonstrated that miR-217 could act on SIRT1 in endothelial cells [125]. Therefore, it’s reasonable to conclude that alcohol-mediated inhibition of SIRT1 in the liver is mediated by miR-217. Additionally, a recent study found that overexpression of miR-217 could induce the expression of lipin-1, which is a critical enzyme catalyzing DAG synthesis and also involved in the development of both alcoholic and non-alcohol fatty liver diseases [126,127,128,129,130]. Therefore, chronic alcohol exposure impairs SIRT1/SREBP-1 axis in a miR-217 dependent manner and ultimately induces hepatic steatosis [124].

Emerging evidence demonstrates that hepatitis virus infection independently facilitates lipid accumulation in the liver, although the exact mechanism is incompletely understood. Hepatitis C Virus (HCV) is an RNA virus responsible for 170 million cases of viral hepatitis worldwide [131]. Nearly half of HCV infected patients develop hepatic steatosis. And expression of HCV core protein alone can trigger fat accumulation in liver [132]. The cytoplasmic lipid droplet (LD) in hepatocytes is crucial for HCV particle assembly, and the host LD scaffold protein perillipin 3 (PLIN3) is reported as an indispensable factor during virus-induced steatosis [133]. As mentioned before, endogenous miRNAs modulate lipid metabolism at post-transcriptionally level, so it is not surprising that the viruses regulate the host miRNAs in multiple ways to facilitate pathogenesis [134]. In recent reports, miR-27, the liver-abundant miRNA described above, is stimulated by HCV both in vitro and in vivo experiments [135]. The upregulation of miR-27 contributes to decreased FFA oxidation and increased FFA uptake, thus facilitates fat accumulation in hepatocyte, which may due to the suppression of its target genes PPARα and ANGPTL3 [136]. ANGPTL3 could inhibit the activity of LPL and further decrease the FFA uptake by the liver. Antagonism of PPARα alone could result in increased cellular triglyceride contents, which is same with the roles of miR-27, while agonist of PPARα reverses miR-27 induced hepatic lipid accumulation [73,135].

In another study, miR-185-5p is decreased by HCV core protein in HEPG2 cells, and meanwhile, SREBP-2, a validated target of miR-185-5P is increased by HCV core protein [136]. SREPB-2 regulates cholesterol homeostasis in the liver through influencing microsomal HMG-CoA reductase (HMGCR), which regulates the rate-limiting step in cholesterol synthesis. The role of HCV core protein in cholesterol metabolism is one of the mechanisms of steatosis induced by HCV infection. This mechanism can be explained by the correlation between miR-185-5p and SREBP2.

## 6. Novel Diagnostic Tools and Treatments for Fatty Liver Diseases

Fatty liver is characterized by excessive triglyceride accumulation as the form of lipid droplets, and liver biopsy is the gold standard to classify different stages of NAFLD. However, there are a number of drawbacks of this procedure, including patient discomfort, a limit value for early diagnosis and the risk for serious complications. Thus, it’s urgent to find noninvasive diagnosis strategies. Currently, ultrasound and other imaging modalities have been applied widely for the diagnosis of NAFLD, but these techniques have failed to distinguish the stage of the disease and to differentiate various causes of NAFLD [137].

As candidate biomarkers for NAFLD, miRNAs are resistant to the degradation by ribonucleases and exist in almost all body fluids. miRNAs could be secreted from the cell via extracellular microvesicles release, and finally detected in peripheral circulation. This kind of miRNAs is termed as circulating miRNAs. The application of circulating miRNAs has been expanded from experimental researches to clinical early disease detection and monitoring of disease progression. For instance, circulating miRNAs were found abnormally expressed in different kinds of cancer. Some specific miRNAs expression patterns have been utilized to distinguish colorectal, pancreatic, hepatocellular carcinoma from normal tissues [138]. As described in this review, miRNAs are closely associated with the pathogenesis of fatty liver disease. Thus, circulating miRNAs may be specific and sensitive biomarkers for fatty liver disease and disease stage assessment.

Recently, a multistage, case-control study was performed to screen a circulating miRNAs profile as diagnose markers for NAFLD [138]. In this study, pooled serum samples from NAFLD patients and healthy volunteers were prepared for high throughput sequencing. Several miRNAs were significantly upregulated in NAFLD samples, like miR-122, miR-27b-3p, miR-192, miR-148a-3p, etc. Among the most abundant miRNAs in the human liver, the increased levels of circulating miRNA-122 in NAFLD has been repeatedly observed in multiple reports [89,139,140]. MiR-122 also accumulates in the serum of ethanol-fed mice model. Furthermore, release of miR-192, miR-30a, miR-14 and miR-155 through exosomes indicates their clinical diagnostic values in alcoholic liver disease [141,142]. It has been reported that miR-122, and miR-34a steadily increased in serum during HCV infection process. Similarly, in a clinical survey, circulation levels of miR-122 and miR-34a are positively correlated with the clinical parameters and stages of disease progression from simple steatosis to steatohepatitis [91].

However, large clinical cohorts are required to clearly confirm circulating miRNAs as a kind of more sensitive and specific biomarker for fatty liver diseases than other noninvasive diagnostic measurements [143,144]. As mentioned previously, miRNAs play important roles in the pathology of liver lipid metabolism, and inappropriate miRNAs expression links to fatty liver disease development. According to that, miRNAs that undergo disease-specific alteration and exhibit cell-specific regulating ability in the liver could be developed as a novel therapeutic target for fatty liver disease. Various studies demonstrated that miRNAs could be new therapeutic targets for many diseases, thus their mimics or antisense oligonucleotides are using to influence the specific endogenous miRNA concentration, subsequently regulating the target genes and biological function [145,146].

However, miRNA-based therapy will face many challenges, including off-target effects, miRNA stability, and binding affinity. But overall a high-efficiency delivery system is a key factor in developing of miRNA-based therapy [42,147,148]. The nanoparticles (NPs) is one of the feasible strategies. Nanoparticles can provide well-organized structural made up of macromolecules which have been designed to carry out biological molecules like peptides, DNA and RNA [149,150]. Assembly of miRNAs into NPs could protect the miRNAs from degradation and enhance their structure stability and circulation time in vivo [151,152]. The small size and capacity for binding the cell-penetrating peptides lead to a huge increase in cellular entry, NPs thereby could also enhance the cellular uptake of miRNAs. However, NPs are foreign molecules and not a part of the host system. After these foreign materials enter into the body, the NPs will be recognized by the immune system and might generate undesirable effects like immune stimulation or suppression. Therefore, the NPs-related immune responses have to be well investigated before as a carrier of miRNAs transport [153].

## 7. Summary

**Figure 4:**
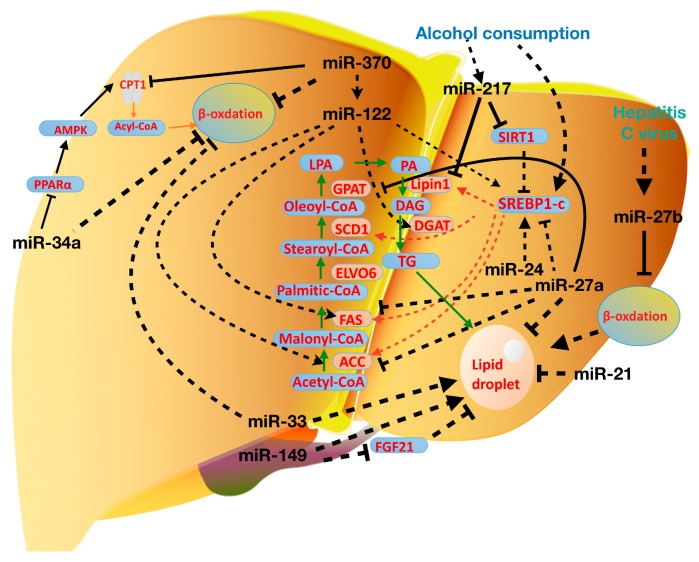
Roles of some miRNAs in fatty liver diseases: miR-122 is a NAFLD related miRNA, which can target many genes in the process of lipogenesis; alcohol consumption stimulate miR-217 expression and then induced lipid accumulation; hepatitis promotes hepatic lipid accumulation through inducing miR-27 expression. The green arrow is used to illustrate the direction of lipogenesis.

Accumulating evidence has suggested that miRNAs are important modulators for lipid metabolism. miRNAs have received much interest not only from a scientific perspective but also their potential clinic applications [154,155,156,157,158]. Our understanding of miRNAs in hepatic lipid metabolism is expanding in Figure 4 and Table 1. However, it is essential at the current stage to clearly define their roles in fatty liver diseases. Since each miRNA has multiple targets and one gene may be regulated by several miRNAs, it is still too early for us to predict any miRNA as a simple target for fatty liver diseases. Nevertheless, with increasing discoveries of miRNAs, once the function of some specific miRNAs in disease pathogenesis is established, we may use these specific miRNAs to diagnose or treat diseases. Finally, it’s reasonable that the clinical applications of miRNAs are certainly promising and hopeful.

## Figures and Tables

**Table 1:** Summary of recently reported miRNA studies in fatty liver disease.

miRNA	Species	Disease-Association	Expression	Target Genes	Findings	Pathologic (+) or Protective (−)	Reference
*miR-122*	Human, mouse	NAFLD	↑ (circulation) ↓	HMGCR, MTTP, HMGCS1PGC1-α	Upregulate the expression of SREBP1-c, DGAT2, FAS and ACC1	+	[37,112,113]
*miR-370*	Human	NAFLD	↑	SREBP-1c, DGAT2	Upregulate the expression of genes involved in lipogenesis Upregulate the expression of miR-122;	+	[39]
*miR-29c*	Human	NAFLD	↑	HMGR, Sirt1	Regulate insulin resistance and lipid metabolism	−	[84]
*miR-216*	Mouse	NAFLD	↓	FAS, SREBP-1c	Regulate the lipid synthesis	/	[61]
*miR-302a*	Mouse	NAFLD	↓	ELOVL; ABCA1	Regulate hepatic lipid accumulation	/	[61]
*miR-34a*	Mouse, Human	NAFLD	↑	PPARα, SIRT1	Decrease FA β-oxidation	−	[68,75,84]
*miR-24*	Human, Mouse	NAFLD	↑	Insig1	Downregulate Insig1 expression; Promote SREBP-1 processing	−	[72]
*miR-21*	Mouse	NAFLD	↓	HMGCR	Regulate liver TG and cholesterol metabolism	+	[76]
*miR-149*	Mouse	NAFLD	↑	FGF21	Regulate lipogenesis in HepG2 cells	−	[77]
*miR-10b*	Human	NAFLD	↑	PPARα	Overexpression of miR-10b increases the triglyceride levels in hepatocytes	−	[82]
*miR-155*	Mouse, Human	NAFLD; Alcoholic fatty liver	↓	LXRα	Regulate LXRα/SREBP-1c signaling and influence liver lipid accumulation.	+/−	[117,118,119,124,125]
*miR-467*	Mouse	NAFLD	↓	LPL	Regulate lipid metabolism through target LPL	+	[86]
*miR-217*	Human	Alcoholic fatty liver	↑	SIRT1; Lipin1	Promote ethanol induced -fat accumulation in hepatocytes	+/−	[133]
*miR-27*	Human	DyslipidemiaVirus hepatitis	↑	PPARα; ANGPTL3	Promote triglyceride accumulation in hepatocytes and inhibit hepatitis C virus replication dyslipidemia animal model	+/−	[60,81,136]
*miR-185-5p*	Human	Virus hepatitis	↓	SREBP2	Regulate cholesterol homeostasis in liver	−	[137]
